# The role of short-chain fatty acids produced by gut microbiota in the regulation of pre-eclampsia onset

**DOI:** 10.3389/fcimb.2023.1177768

**Published:** 2023-07-12

**Authors:** Jinghan Cui, Jun Wang, Ying Wang

**Affiliations:** Department of Obstetrics and Gynecology, Shengjing Hospital of China Medical University, Shenyang, China

**Keywords:** short-chain fatty acid, preeclampsia, gut microbiota, gestational diseases, pathogenesis of preeclampsia, immunocyte, inflammatory response

## Abstract

**Background:**

Preeclampsia (PE) is a common pregnancy-related disorder characterized by disrupted maternal-fetal immune tolerance, involving diffuse inflammatory responses and vascular endothelial damage. Alterations in the gut microbiota (GM) during pregnancy can affect intestinal barrier function and immune balance.

**Aims and purpose:**

This comprehensive review aims to investigate the potential role of short-chain fatty acids (SCFAs), essential metabolites produced by the GM, in the development of PE. The purpose is to examine their impact on colonic peripheral regulatory T (Treg) cells, the pathogenic potential of antigen-specific helper T (Th) cells, and the inflammatory pathways associated with immune homeostasis.

**Key insights:**

An increasing body of evidence suggests that dysbiosis in the GM can lead to alterations in SCFA levels, which may significantly contribute to the development of PE. SCFAs enhance the number and function of colonic Treg cells, mitigate the pathogenic potential of GM-specific Th cells, and inhibit inflammatory progression, thereby maintaining immune homeostasis. These insights highlight the potential significance of GM dysregulation and SCFAs produced by GM in the pathogenesis of PE. While the exact causes of PE remain elusive, and definitive clinical treatments are lacking, the GM and SCFAs present promising avenues for future clinical applications related to PE, offering a novel approach for prophylaxis and therapy.

## 1 Introduction

Preeclampsia (PE) is a pregnancy-specific disorder characterized by hypertension (Mol et al., 2016). It is a subtype of metabolic disease, with most cases occurring in late pregnancy, while early onset is associated with worse prognosis (Malik and Kumar, 2017). The causes of PE remain unknown, but potential pathogenic factors may involve thrombophilia, inflammation, oxidative stress, and genetic variants of the renin-angiotensin system (Goetzinger et al., 2014; Mol et al., 2016; Rana et al., 2019). Recently, some researchers have hypothesized that changes in the microbiota, microbial metabolites, and their interactions with the immune and endocrine systems may be associated with various diseases (Gilbert et al., 2018) that can lead to obesity, elevated blood pressure, and other comorbidities, which in turn increase the probability of illness (Poon et al., 2019; Ishimwe, 2021). The gut microbiota (GM) plays a role in maintaining intestinal and immune homeostasis through processes like carbohydrate digestion and the production of metabolites. However, disruptions in the stable relationship between the host and its microbiota, known as GM dysregulation, can lead to immune system disorders and the development of diseases, such as hypertension, obesity, and diabetes (Delhaes et al., 2018; Robles-Vera et al., 2020; Jin et al., 2022). However, the mechanisms by which the GM influences the progression of PE have not been fully understood to date.

In 2012, Omry et al. conducted a pioneering study investigating changes in GM and their association with metabolic disorders. They observed a significant alteration in GM composition, i.e., reduced diversity, in women during the third trimester of pregnancy compared to early pregnancy. Additionally, the progression of pregnancy was found to lead to low-level inflammation on the gut mucosal surface, indicated by elevated levels of pro-inflammatory cytokines and leukocytes, which in turn could contribute to GM disorders (Lv et al., 2019; Wang et al., 2020). Moreover, when GM from women in the third trimester was transferred to germ-free mice, metabolic changes such as significant fat accumulation and insulin resistance were induced, suggesting the relevance of GM to inflammatory metabolic diseases (Koren et al., 2012). In a study by Chen et al., third trimester PE patients, along with normotensive pregnant women, were recruited. The researchers observed that when the GM of patients was transferred to germ-free mice, the mice developed PE-like symptoms, including elevated blood pressure and increased urinary protein (Liu et al., 2017; Yong et al., 2022). These findings suggest a potential correlation between GM dysregulation and the development of PE (Huang et al., 2021). Further analysis comparing splenocytes and lymphocytes in the lamina propria of the small intestine using flow cytometry showed a significant decrease in Treg cells and a significant increase in Th17 cells in PE patients (Eghbal-Fard et al., 2019), suggesting that GM may play a role in the pathogenetic progression of PE by disrupting mucosal and systemic immune responses (Pickard et al., 2017; Chen et al., 2020).

**Figure 1:**
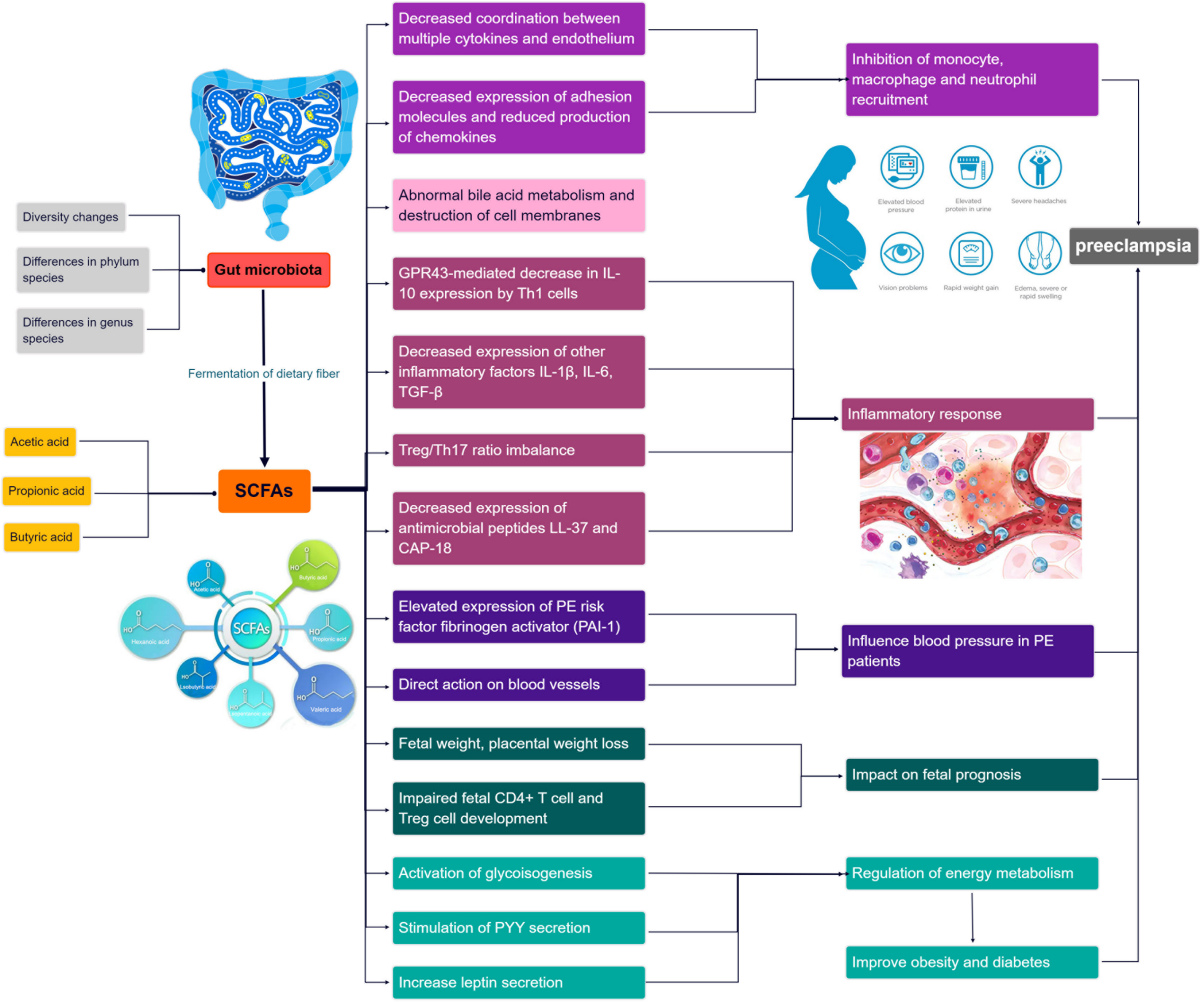
Overview of the role and relevance of GM and SCFAs in the pathogenesis of PE progression.

In recent years, emerging research has highlighted the potential of short-chain fatty acids (SCFAs) (Dalile et al., 2019), a prominent metabolite of the gut microbiota (GM), in the progression of GM-associated PE (Jin et al., 2022). Smith et al. conducted a study demonstrating that SCFAs can restore the number of colonic regulatory T cells in GM-deficient mice, indicating their influence on Treg cell function and immune modulation (Smith et al., 2013). Moreover, SCFAs have been shown to regulate cytokine expression in macrophages through G protein-coupled receptors (GPCRs), promoting M2 macrophage polarization and inhibiting lipopolysaccharide (LPS)-induced M1 macrophage polarization, ultimately reducing the inflammatory response and acting as anti-inflammatory agents (Ji et al., 2016; Jiang et al., 2020). Furthermore, Jin et al. conducted experiments in PE rats and found that oral administration of propionic acid or butyric acid significantly lowered blood pressure, improved placental function, and enhanced embryonic development, suggesting a potential therapeutic role for SCFAs in PE (Jin et al., 2022). The dysregulation of SCFAs resulting from GM alterations may contribute to the pathogenesis of PE by affecting various immune cells. The roles of GM and SCFAs in the pathogenesis of PE progression are summarized in **Figure 1**. Exploring the specific mechanisms and roles by which SCFAs regulate PE pathogenesis in the context of GM dysregulation may provide a promising avenue for the prevention and treatment of PE.

## 2 Methods

The aim of this narrative review is to explore the role played by GM in PE progression and the relationship between SCFAs produced by GM metabolism and PE, respectively, in terms of anti-inflammatory effects, reduction of GDM, and PE comorbidities such as obesity and hypertension. A literature search was conducted using the PubMed/Web Of Science database to retrieve relevant English language studies published in the past 20 years. The authors independently identified the most relevant studies, including randomized controlled trials (RCTs) (**Table 1**), experimental and observational studies. The inclusion criteria focused on RCTs, human cohort studies, and *in vivo/in vitro* studies with clear experimental methods and relevant outcomes. Studies with unclear methods, experimental subjects, or negative/ambiguous results were excluded. The search utilized keywords such as gut microbiota, intestinal inflammation, PE, gestational hypertension, hypertension, gestational diabetes, obesity, diabetes, SCFAs, metabolism, pregnancy and gestational disorders.

**Table 1:** Summary of controlled experiments on the role of SCFAs and GM in regulating PE progression.

Author	Year	Experimental Model	Outcome	Result	Ref.
Human cohort-controlled trial					
Chang	2020	27 severe PE patients and 36 pregnant women with normal pregnancies	Comparison of GM abundance of intestinal flora and its relationship with SCFAs.	There was a consistent correlation between SCFAs and bacterial GM abundance in PE patients. The phylum Thick-walled and its subordinates were associated with increased SCFAs, whereas the phylum Aspergillus and its subordinate Escherichia_Shigella were associated with decreased SCFAs. All SCFAs were synchronously correlated with GM abundance differences, suggesting that GM abundance differences and the SCFAs they produce may play a synchronous role in the development of PE. Within the SCFAs, butyric and valeric acid were most strongly correlated with GM abundance differences.	(Chang et al., 2020)
Jin	2022	92 PE patients and 86 pregnant women with normal pregnancies	The variation of GM abundance in intestinal flora, its relationship with SCFAs, and its mechanism of action.	SCFAs can regulate the expression of a wide range of cytokines in macrophages *via* GPCRs, which have anti-inflammatory functions. For example, butyric acid promotes M2-type macrophage polarization and inhibits LPS-induced M1-type macrophage polarization, and also enhances autophagy, thereby inhibiting macrophage-induced inflammation and significantly improving PE symptoms in rats.	(Jin et al., 2022)
Hu	2019	Cohort 1: 50 patients with PE and 50 normal pregnant women. Cohort 2: 887 pregnancies in the second trimester, 24 of which developed PE in the late pregnancy.	Fetal thymus volume and diameter in cohort 1 of two groups. Relationship between serum SCFAs content and subsequent PE in Cohort 2 at 28 weeks of gestation.	In cohort 1, fetal thymus volume and fetal thymus diameter were reduced by 38% and 12.1% in patients with PE compared with normal pregnancy. Patients with PE in cohort 2 were found to have serum acetate levels at least ten times higher than those of butyrate and propionate, and the relative risk of PE was reduced for every 30% increase in serum acetate. In addition, maternal supplementation with acetate returned the overall amount of CD4+ T cells in PE-FMT mice pups to the normal control total, and acetate supplementation increased Foxp3+ expression in Treg cells.	(Hu et al., 2019)
Altemain	2021	11 PE patients and 22 pregnant women with normal pregnancies.	Composition of the GM in patients with DPE at 28 weeks of gestation. Assessment of the density of butyrate producing genes.	A trend towards significantly lower serum acetate, propionate and butyrate concentrations was found in patients with DPE, increased fecal SCFAs in normal controls and a significant decrease in butyrate in PE. And there was a negative association between acetate and Coprococcus abundance in women with DPE. A significant negative association was also found between fasting triglyceride levels and total butyrate levels in pregnant.	(Altemani et al., 2021)
Gomes	2016	205 pregnant women at 16 weeks’ gestation.	GM composition and levels of SCFAs in relationship to PAI-1 concentration and blood pressure in obese pregnancies.	In overweight and obese pregnancies at 16 weeks of gestation, GM abundance of butyrate production was negatively and significantly correlated with PAI-1 levels and blood pressure, whereas circulating PAI-1 was elevated in hypertensive patients. In addition, SCFAs were found to expand isolated human colonic arteries *in vitro*, and oral administration of butyrate reduced altered concentrations of IL-1β and TNF-α in mice induced by a high-fat diet.	(Gomez-Arango et al., 2016)
Tang	2022	6 patients with PE and 8 pregnant women with normal pregnancies.	Changes in the abundance of GM and the relationship between GM of PE and lncRNA.	The LPS levels in stool and placenta in PE group were significantly higher than those in control group, and the circulating lncRNABC030099 levels in plasma were also significantly higher than those in healthy subjects. The effect of GM on host lipid metabolism may be mediated by metabolites produced by GM, including SCFAs, secondary bile acids and trimethylamine, as well as LPS.	(Tang et al., 2022)
Huang	2021	Group NW: 21 normal women; Group NP: 28 normal pregnancies; Group APG: 25 pregnancies having decreased PIGF concentration; Group PE: 26 PE patients.	The correlation between GM and PE progression, and whether PIGF can predict placental abnormalities associated with PE.	Compared with healthy pregnant women, GM composition in group PE and group APG was significantly changed. At the genus level, a clear uneven distribution of GM abundance can be noticed, particularly in the genera with positive LDA score by linear discriminant analysis. The beneficial bacteria Lactobacillus was significantly reduced in the groups PE and APG, but it was only associated with blood pressure and albuminuria levels in the group PE.	(Huang et al., 2021)
Experiments on animals					
Robles	2020	10 five-week-old Wistar Kyoto rats (WKY) and fifty SHR rats	SHR rats were orally treated with butyrate or acetate to observe its cardiovascular effects.	Treatment with acetate and butyrate inhibited the development of hypertension and normalized Firmicutes and Bacteroides (F/B) ratios. In addition, Th17 cell/Treg cell balance in mesenteric lymph nodes was restored, endotoxemia was normalized, and mRNA expression levels of tight junction protein occlusive protein and ZO-1 in colon were increased.	(Robles-Vera et al., 2020)
Yong	2022	15 normal pregnant rats, 15 PE model group, 15 PE model sodium butyrate treated group.	The influence of administering sodium butyrate on the progression of PE.	Sodium butyrate significantly reduced host blood pressure, inflammatory factor expression and urinary protein levels, and increased placental weight as well as expression levels of placental growth factor and gut barrier markers. Also, sodium butyrate treatment decreased the Treg/Th17 cell ratio of spleen and small intestine immune cells in pregnant rats, and also improved the abundance of Bacteroides and Firmicutes.	(Yong et al., 2022)
Onyszkiewicz	2019	4-16 weeks male Wistar rats.	Butyric acid may exert hemodynamic effects *via* intestinal signaling to lower blood pressure.	A 2-3-fold increase in butyric acid concentration in the colon produces a significant hypotensive effect, which appears to be mediated by colonic afferent nerve signaling and GPR41/43 receptors. It may also involve vasodilation induced by hematogenous butyric acid.	(Onyszkiewicz et al., 2019)
Smith	2013	SPF mice, GF mice	SCFAs regulates the size and function of colonic Treg cell pools and how to prevent colitis in mice.	It was found that the concentration of SCFAs in the colonic cavity of GF mice was decreased, and the expression of Foxp3 and IL-10 was increased after treatment with propionate. When treated with a combination of vancomycin and SCFAs, the reduction of Treg cells was completely restored. By constructing a model of T cell transfer in colitis, it was found that the frequency and number of Foxp3+Treg cells increased in mice receiving the propionic acid and SCFAs mixture.	(Smith et al., 2013)
Sun	2018	C57BL/6 and Rag-/- mice, construction of colitis model mice	How SCFAs regulates Th1 cell function.	SCFAs promotes the production of IL-10 by GM-specific Th1 cells, which is mediated by GPCR43, as a way to reduce Th1 cell-induced gut inflammation. The mechanism is dependent on the mTOR and STAT3 activation, thus promoting the expression of the transcription factor Blimp-1 in Th1 cells.	(Sun et al., 2018)
Hsu	2018	Pregnant SD rats	To investigate whether early GM-targeted therapy with probiotic Lactobacillus casei and prebiotic inulin can prevent high-fructose (HF) diet-induced programmed hypertension.	Prebiotic treatment to prevent HF-induced hypertension was found to be associated with reduced plasma acetate levels and reduced renal mRNA expression, whereas probiotic treatment increased plasma propionate levels and restored the HF-induced reduction in FAR2 (Fatty Acyl-CoA Reductase 2) expression. Maternal HF diet has long-term programming effects on GM in adult offspring. Maternal GM-targeted therapies may be influencing the progression of hypertension by reprogramming and, in turn, influencing the progression of hypertension.	(Hsu et al., 2018)
Marques	2017	Male C57BL/6 mice.	Whether a high-fiber diet and acetate supplementation may play a protective role in cardiovascular disease.	Studies have shown that high fiber intake alters GM and increases the abundance of acetate producing bacteria, and that acetate improves intestinal ecological dysregulation by altering the ratio of Firmicutes to Bacteroides. Acetate supplementation significantly reduced systolic and diastolic blood pressure, cardiac fibrosis and left ventricular hypertrophy in hypertensive mice.	(Marques et al., 2017)
Kim	2018	Adult C57BL6 mice and Sprague–Dawley (SD) rats	Validating whether hypertensive patients have different GM and whether GM markers can predict hypertension	Plasma concentrations of intestinal fatty acid binding protein (I-FABP), LPS, and intestinal-targeted pro-inflammatory Th17 cells were found to be significantly increased in hypertensive patients, indicating increased intestinal inflammation and permeability, suggesting that intestinal barrier dysfunction and microbiome function are associated with human hypertension.	(Kim et al., 2018)
Bartolomaeus	2019	Wild-type NMRI or apolipoprotein E knockout-deficient mice.	To study the effects of SCFAs on cardiac injury and atherosclerosis in hypertensive mice.	In both models, propionate significantly attenuated cardiac hypertrophy, fibrosis, vascular dysfunction and hypertension and significantly reduced susceptibility to ventricular arrhythmias, as well as reduced the area of aortic atherosclerosis. Propionate treatment reduced systemic inflammation as evidenced by a reduction in splenic effector memory T cell frequency and splenic Th17 cells in both models.	(Bartolomaeus et al., 2019)

## 3 Relationship between SCFAs and GM alterations

### 3.1 GM metabolism produces SCFAs

SCFAs are major metabolites produced through the fermentation of carbohydrates by the GM and are important for maintaining gut homeostasis (Parada Venegas et al., 2019). They have several beneficial effects in the human body (Macfarlane and Macfarlane, 2003; Yan and Ajuwon, 2015), including improving gut hormone secretion, regulating inflammatory homeostasis and the composition of GM, as well as having protective effects on gastrointestinal barrier function (Lee et al., 2017), as well as regulating blood pressure by affecting epithelial, nervous, immune system, and vascular functions. It is speculated that SCFAs may affect the progress of PE. SCFAs are saturated aliphatic organic acids consisting of one to six carbons, of which acetate, propionate, and butyrate are the most abundant (≥95%). The molar ratio of propionate, butyrate, and acetate in the colon and feces is approximately 20:20:60, and a series of postmortem examinations conducted on individuals who passed away unexpectedly (n = 6) indicated that the ratios of propionate, butyrate, and acetate were similar in the proximal and distal zones of the large intestine (Cummings et al., 1987). In the cecum and large intestine, approximately 95% of SCFAs are quickly taken up by colonic cells, with the other 5% excreted in the feces (Ruppin et al., 1980; Den Besten et al., 2013). The main butyrate-producing bacteria in the human gut belong to the phylum Firmicutes, including the genera Clostridium faecium and Eubacterium. Members of other phyla, especially the Bacteroidetes like Odoribacter and Alistipes, primarily produce acetate and propionate (Duncan et al., 2002; Gomez-Arango et al., 2016).

**Figure 2:**
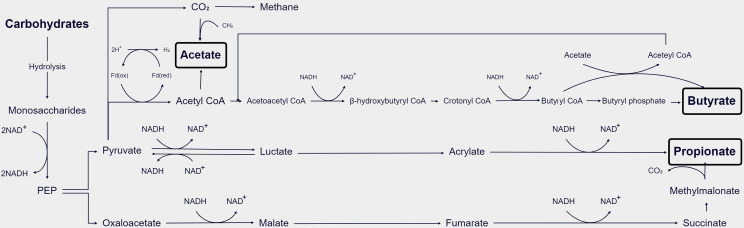
Schematic diagram of the process of GM metabolizing SCFAs in the body (Den Besten et al., 2013).

GM hydrolyzes carbohydrates into monosaccharides, which are subsequently fermented in the gut’s anaerobic environment. The main metabolic pathways of bacteria include the glycolytic pathways for the six carbon sugars (Embden–Meyerhof–Parnas pathway) and the five carbon sugars (pentose phosphate pathway), which transform monosaccharides into phosphoenolpyruvate (PEP). Thereafter, PEP is transformed into various products of fermentation, like organic acids or alcohols. During the glyceraldehyde-3-phosphate dehydrogenase (GAPDH) step, reduced coenzyme I (nicotinamide adenine dinucleotide, NADH) is produced. Under anaerobic conditions, excess reducing equivalents can be eliminated through the following mechanisms (**Figure 2**). In the first classical fermentation pathway, NADH is oxidized to reduce pyruvate to lactic acid or ethanol. Additionally, the pathway generates molecular hydrogen (H2) from the excess reducing equivalents through the pyruvate pathway or the endomembrane pathway (Fischbach and Sonnenburg, 2011). The main end products of the above fermentation pathway are SCFAs (Den Besten et al., 2013) (**Figure 2**). Acetate can be formed directly from acetyl-coenzyme A (acetyl-CoA) or *via* the Wood–Ljungdahl pathway, which involves the utilization of formate (Ragsdale and Pierce, 2008). Propionate can be formed *via* the succinate decarboxylation pathway or through the conversion of PEP *via* the acrylate pathway (Miller and Wolin, 1996). The condensation of two acetyl-CoA molecules produces butyrate *via* butyrate kinase (buk) or using butyryl-coenzyme, acetate-coenzyme A transferase to produce butyrate using exogenously derived acetate (Duncan et al., 2002).

### 3.2 Effect of altered GM abundance of PE on SCFAs

Altemani et al. discovered that patients with late-onset preeclampsia (DPE), specifically those with >34 weeks of gestation, exhibited reduced serum levels of butyric acid. Furthermore, a decrease in the gene abundance of butyryl-coenzyme A, acetate-coenzyme A transferase, and buk was observed in the GM, indicating a correlation between reduced levels of butyric acid produced by the GM and PE (Altemani et al., 2021; Li et al., 2022). Additionally, alterations in the composition of GM species are associated with the pathophysiological and immune status of patients, and these changes are significantly correlated with SCFA levels (Yan et al., 2017; Ahmadian et al., 2020; Jin et al., 2022). Studies isolating bacteria from human clarified rumen fluid and fecal samples have identified major butyrate producers in the human intestine, including members of the Firmicutes phylum, such as Clostridium, Faecalibacterium from the Rumenobacteriaceae family, and Eubacterium Rectale from the Lachnospiraceae family (Louis and Flint, 2017). Other bacteria, such as Actinomycetes, Bacteroides, Clostridium, Proteus, and Thermotogae, may also have the capacity to produce butyrate depending on the genes they express (Duncan et al., 2002; Gomez-Arango et al., 2016). Within the Bacteroidetes phylum, Odoribacter and Alistipes species are known to primarily produce acetate and butyrate (Macfarlane and Macfarlane, 2003). Bifidobacterium, belonging to the Actinomycetes phylum, is mainly responsible for acetate and lactic acid production, while the mucin-degrading bacterium Akkermansia muciniphila can produce propionate and acetate (Parada Venegas et al., 2019).

The researchers utilized fecal specimens from severe PE patients and pregnant healthy controls to assess the abundance of GM and the content of fecal SCFAs through 16SrRNA sequencing and gas chromatography. Heat maps were generated to visualize the correlations between GM abundance differences and SCFAs levels based on the data results. Analysis revealed that the most abundant SCFAs in the feces were acetic, propionic, and butyric acid, while valeric, isovaleric, and isobutyric acids constituted a smaller fraction of the total SCFAs. Remarkably, reduced concentrations of butyric and valeric acids were observed in individuals with PE (Chang et al., 2020).

On the genus level, several bacteria showed positive correlations with fecal SCFAs levels, particularly pentanoic and butyric acids. These bacteria included Alistipes, Blautia, Collinsella, Eubacterium Rectale, and Streptococcus, belonging to the phyla Actinomycetes, Bacteroidetes, or Firmicutes (Yu et al., 2018; Chang et al., 2020). Notably, a negative association was found between systolic blood pressure and the abundance of Eubacterium Rectale and Blautia in the gut, indicating a decrease in the relative abundance of these bacteria in hypertensive patients (Saji et al., 2019; Toral et al., 2019a). Conversely, bacteria such as Escherichia, Shigella (both belonging to Proteobacteria), and Enterobacter (also belonging to Proteobacteria) exhibited negative associations with fecal concentrations of acetic, propionic, butyric, and valeric acids. It has been reported that Escherichia and Enterobacter, enriched in the gut of PE patients, can lead to excessive inflammatory responses (Reuschel et al., 2020). These findings suggest that changes in fecal SCFA levels align with variations in GM abundance in the PE group (Chang et al., 2020).

Additionally, Altemani et al. identified bacterial species associated with butyrate production that were present in low numbers at 28 weeks of pregnancy, before the onset of PE symptoms. This reduction in specific butyrate-producing bacterial species was observed to persist in individuals who later developed PE. The study also found significantly lower levels of butyrate in the blood of individuals with late-onset preeclampsia compared to the control group. These findings suggest that a decrease in the relative abundance of specific bacterial species involved in butyrate production may contribute to the risk of developing PE in the third trimester of pregnancy, particularly in obese pregnant women (Altemani et al., 2021).

## 4 Correlation between GM changes and PE

### 4.1 Inflammatory response due to maternal GM alterations and effects on fetal health

**Figure 3:**
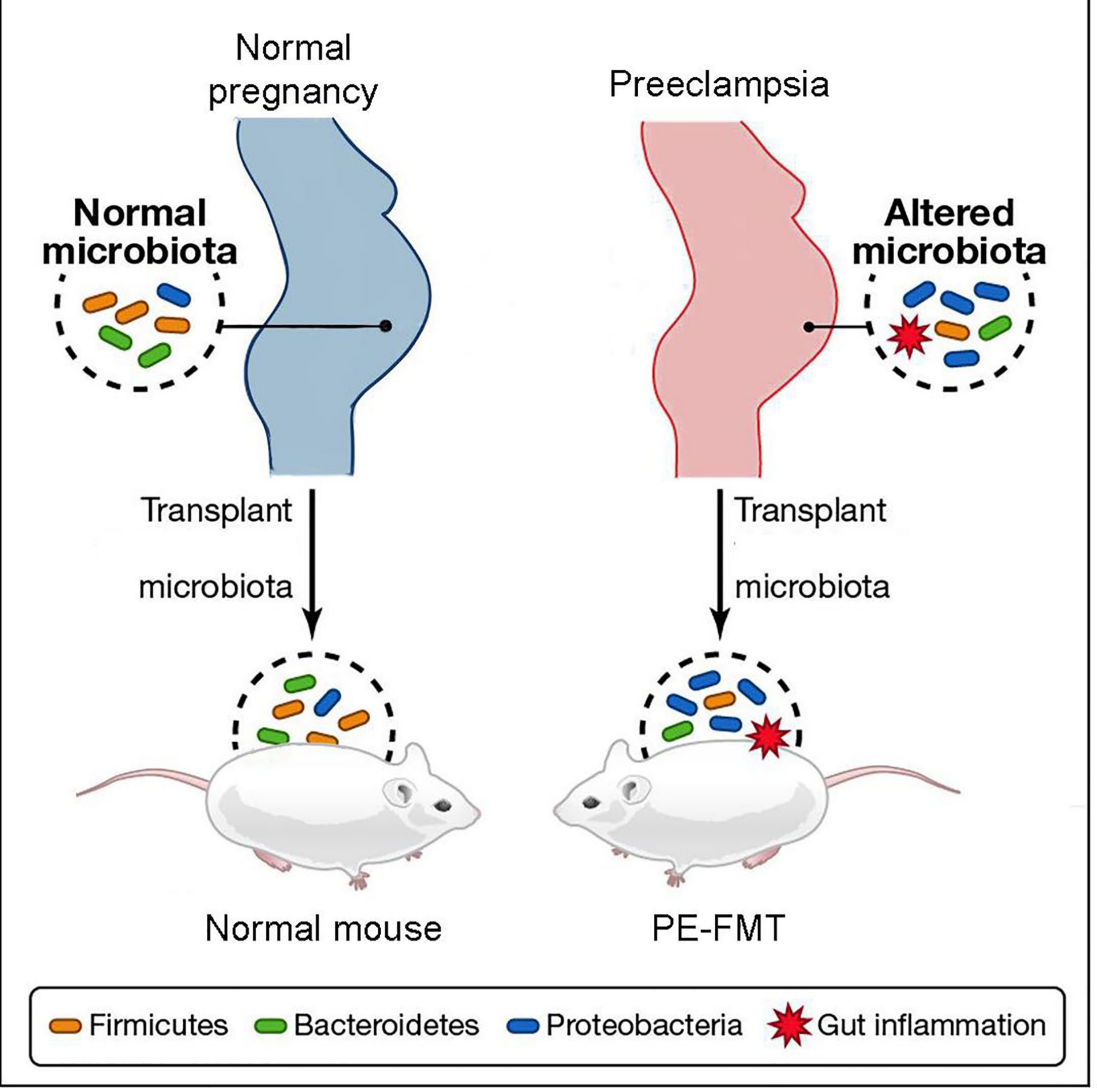
Schematic diagram of fecal microbiota transplantation.

Through the collection of stool samples from PE patients and healthy individuals (2020), researchers conducted 16S rRNA sequencing and discovered notable differences in the abundance of Bacteroidetes, Proteobacteria, and Fusobacterium in PE patients, indicating an altered microbial diversity between the two groups (Wang et al., 2019; Meijer et al., 2023). Additionally, PE patients exhibited a significant increase in inflammatory factors, including IL-6 and TNF-α, in their plasma (Black and Horowitz, 2018; Zhao et al., 2022). These findings suggest that patients with PE experience a characteristic activation of the inflammatory response (Lavelle and Sokol, 2020). Fecal microbiota transplantation experiments (**Figure 3**) demonstrated that this inflammatory response is linked to GM alterations (Kim et al., 2017; Michaudel and Sokol, 2020). The utilization of fecal microbiota transplantation (FMT) from PE patients in an antibiotic-treated mouse model triggered a PE-like phenotype in mice (PE-FMT mice). Translocation of microbiota and immune alterations in human and mouse placental specimens can be identified by 16SrRNA sequencing and quantitative PCR (Chen et al., 2020). PE-FMT mice exhibited decreased Treg cell number and function and increased Th17 cells (Darmochwal-Kolarz et al., 2012; Eviston et al., 2012). In a rat model of PE with reduced uterine perfusion pressure (RUPP), chronic inflammation was found to be associated with an imbalance between T cell subtypes (Cornelius et al., 2016). Much of the work on GM and immunity in PE patients is focused on imbalances in T cell homeostasis when GM, through interaction with T cells, results in impaired gut barrier function and microbial molecular translocation. However, T cells may also be involved in the peri-implantation period, impacting both the embryo and mother (Hu et al., 2019).

Furthermore, the inflammatory response mediated by the GM is not limited to specific bacterial species but also involves other bacterial metabolites, such as endotoxin and LPS, which possess high inflammatory potential (Robles-Vera et al., 2018). These molecules can activate the innate immune response, thereby exacerbating the overall inflammatory effects (Kell and Kenny, 2016; Armani et al., 2017). The sustained presence of such inflammatory stimuli can contribute to chronic inflammation (Kim et al., 2017), leading to an increase in inflammatory cytokines, oxidative stress in the placenta, impaired vascular function, and ultimately the progression of fetal immune rejection and hypertension (Chen et al., 2020).

Moreover, emerging evidence suggests that alterations in maternal GM can influence the composition of the fetal microbiota, and the maternal gut environment may have long-term effects on the health of offspring through mechanisms such as developmental programming (Alexander, 2006; Torres et al., 2020). Studies in animal models have demonstrated that prenatal modulation of maternal microbiota through the administration of probiotics or prebiotics can confer protection against cardiovascular diseases, including hypertension, in the offspring (Hsu et al., 2018). Additionally, maternal GM has been shown to shape the immune system of offspring, as pups born to pregnant mice with transiently modified GM during pregnancy exhibit enhanced ability to modulate inflammatory responses to GM (Gomez De Agüero et al., 2016; Koch et al., 2016). Disruption of maternal GM can also impact fetal immune mechanisms, potentially leading to immune disorders such as asthma (Zhao et al., 2015; Nyangahu et al., 2018). Collectively, these findings suggest that GM may play a role in the progression of PE and have implications for fetal health by influencing immune homeostasis and triggering inflammatory responses.

### 4.2 Association of GM alterations with common PE comorbidities hypertension, obesity, and diabetes

PE is characterized by hypertension and studies have indicated a potential association between blood pressure and the GM (Yang et al., 2018; Tang et al., 2019). Transplantation of fecal material from hypertensive human donors into germ-free mice has shown that bacterial transfer can induce an increase in blood pressure in the recipient mice (Muralitharan et al., 2020), suggesting a direct impact of GM on host blood pressure regulation (Yang et al., 2015; Li et al., 2017). Several mechanisms have been proposed to explain how GM may influence blood pressure in the host. Certain bacteria, such as Bifidobacterium, Escherichia, Lactobacillus and Streptococcus have, been implicated in neurotransmitter synthesis within the autonomic nervous system, and alterations in their abundance can consequently affect vascular tone and contribute to the development of hypertension (El Aidy et al., 2014). GM can also influence blood pressure by modulating inflammatory reactions in the body, affecting vascular endothelial function, and subsequently influencing blood pressure regulation (Gomez-Arango et al., 2016). Furthermore, GM can produce bioactive metabolites, including trimethylamine (TMA), trimethylamine N-oxide (TMAO) and SCFAs (Tang et al., 2019). TMAO, GM-derived metabolite associated with increased levels of choline and carnitine in the gut, has been found to be significantly elevated in the peripheral circulation of PE patients (Cornelius et al., 2016). Observational studies have suggested that TMAO may contribute to the development of cardiovascular disease (Wang et al., 2019). Additionally, Koeth et al. demonstrated that TMAO plays a significant role in atherosclerosis progression by regulating sterol and cholesterol metabolism within the host (Koeth et al., 2013), and atherosclerotic-like lesions have been observed in the placenta of PE patients, characterized by lipid deposition in the walls of spiral arteries (Hu et al., 2019). Therefore, it is hypothesized that TMAO might accelerate the formation of atherosclerotic lesions, thus contributing to the development of PE (Wang et al., 2019). These results support a potential correlation between GM dysregulation and elevated blood pressure.

Numerous studies conducted on both human cohorts and animal models have demonstrated alterations in GM abundance and diversity in the context of obesity (Gérard, 2016). Ley et al. reported a significant increase in Firmicutes and a decrease in Bacteroides in obese mice compared to non-obese mice (Ley et al., 2005). Collado et al. conducted a comparison of GM composition between obese and healthy weight pregnant women using qPCR and other techniques, revealing a significant increase in Bacteroides and Staphylococcus in overweight and obese females (Collado et al., 2008). Furthermore, an elevated number of Staphylococcus was positively correlated with increased plasma cholesterol levels, which could be attributed to the production of distinct SCFAs and the modulation of host gene expression influencing lipid metabolism (Pouteau et al., 2003; Turnbaugh et al., 2006). In an experiment conducted on SD rats, prebiotic treatment selectively modified the GM composition of obese dams and enhanced the secretion of intestinal satiety hormones such as glucagon-like peptide-1 (GLP-1) (Bell et al., 2005). This resulted in a reduction in maternal energy intake, a decrease in body weight gained during pregnancy, and a decrease in the probability of excessive weight gain in the mother and her offspring (Paul et al., 2016). Additionally, GM alterations have the potential to predict changes in metabolic pathways such as glycolysis and lipid metabolism (Gohir et al., 2015). Through 16S rRNA sequencing of fecal samples from individuals with gestational diabetes and normal pregnancies, it was discovered that Akkermansia was associated with lower systemic insulin sensitivity (Hansen et al., 2012). In rodent studies, supplementation with the probiotic Akkermansia improved glucose tolerance and insulin sensitivity (Zhao et al., 2017; Crusell et al., 2018). These findings indicate the potential metabolic benefits of probiotics, including improvements in obesity, hypertension, and diabetes, which are commonly observed comorbidities of PE. Therefore, probiotic interventions may also hold promise for mitigating the progression of PE.

## 5 Mechanism of action of SCFAs in PE

### 5.1 Anti-inflammatory effect of SCFAs in the progression of PE

**Figure 4:**
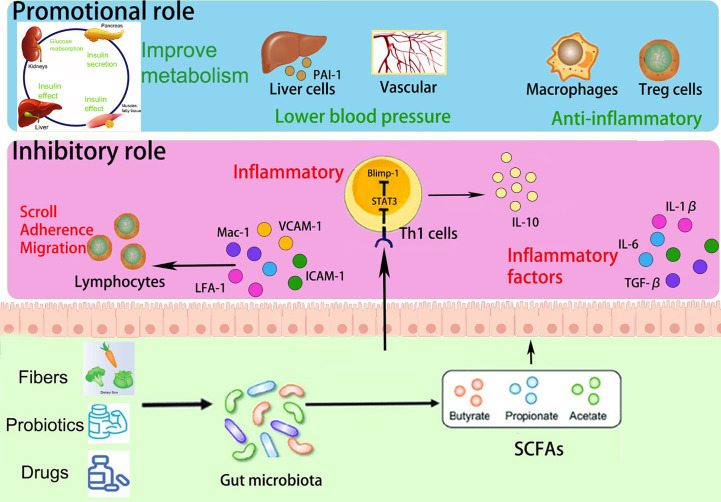
Promotional and inhibitory effects of SCFAs in PE progression.

SCFAs play a multifaceted role in immune regulation. They not only affect the production of interleukins and leukocyte migration but also induce apoptosis in macrophages, lymphocytes (Bailón et al., 2010), and neutrophils (Vinolo et al., 2011). Additionally, SCFAs can inhibit the expression of adhesion molecules induced by stimuli and the production of chemokines, as well as their interactions with endothelial cells. This includes selectins, integrins (such as macrophage antigen-1 [Mac-1] and lymphocyte function-associated antigen-1 [LFA-1]), anti-ligand molecules like intercellular adhesion molecule-1 (ICAM-1), specific carbohydrates, and vascular cell adhesion molecule-1 (VCAM-1) (Vinolo et al., 2009) (**Figure 4**). These cytoadhesion factors and chemokines play crucial roles in leukocyte rolling along vascular endothelium, stable adhesion, and trans-endothelial migration (Meijer et al., 2010). SCFAs can suppress the recruitment of neutrophils, monocytes, and macrophages through the aforementioned cytokines, exerting anti-inflammatory effects. Furthermore, SCFAs also have the ability to regulate gut cell renewal, maintain barrier function, and modulate responses to inflammatory or infectious stimuli, thus playing an essential role in intestinal epithelial physiology (Corrêa-Oliveira et al., 2016).

Among the metabolites produced by the fermentation of dietary fiber by gut microbiota, SCFAs also include the pro-inflammatory metabolite lactic acid. The inflammatory effects of lactic acid are associated with bile acids, and disturbances in bile acid metabolism can lead to abnormal inflammatory responses. Certain bile acids, due to their hydrophobic properties, can disrupt cell membranes (Hang et al., 2019). Consequently, the resulting increase in lactate levels has been shown to be associated with hypertension and inflammatory response (Durgan et al., 2016; Ishimwe et al., 2021).

Furthermore, Yu et al. constructed a mouse model utilizing the T-cell over-transfer colitis method, whereby Th1 cells or naive CD4+ T cells were injected intravenously into mice and subjected to butyrate treatment and flow cytometry analysis. SCFAs were shown to promote IL-10 generation by GM antigen-specific Th1 cells, a process mediated through GPR3 (Sun et al., 2018). Mechanistically, this relies on the activation of STAT3 and mTOR, which promote the expression of the transcription factor Blimp-1 in Th1 cells. Consequently, SCFAs attenuate GM-specific Th1 cell-induced inflammation in the intestine (Heinemann et al., 2014; Sun et al., 2018) (**Figure 4**). In addition, the offspring of PE patients have been found to exhibit persistent alterations in fetal thymus size and structure and reduced output of thymic regulatory T cells, which can be observed until the age of 4 years (Sun et al., 2018). In studies using germ-free mice, researchers observed that the growth and development of fetal thymus Treg cells and CD4+ T cells were similarly impaired, and this impairment could be reversed by maternal supplementation with acetate. The administration of acetate, one of the SCFAs, induced an increased expression of autoimmune regulator (AIRE), a transcription factor involved in facilitating Treg cell production (Heinemann et al., 2014). These findings suggest that maternal acetate supplementation may have the potential to pass through the placenta and influence fetal T cell immunity, thereby impacting the altered development of T cells and immune function observed in the offspring of PE patients (Hu et al., 2019).

Moreover, Yong et al. induced a PE-like phenotype in pregnant rats using Nω-nitro- L-arginine methyl ester hydrochloride, and found that sodium butyrate significantly decreased blood pressure, 24-hour proteinuria, and the expression levels of inflammatory factors including TGF-β, IL- 1, and IL- 6, as well as increasing placental weight and expression of gut barrier markers such as claudin-5, ocludin, and ZO-1 (Chang et al., 2020; Yong et al., 2022). Meanwhile, a decrease in Treg/Th17 cell ratios and an increase in pregnancy-associated dimethylamine oxidase levels in spleen and small intestine immune cells were also observed in sodium butyrate-treated pregnant rats. In addition, sodium butyrate increased the expression of antimicrobial peptides such as LL-37 and CAP-18, in rabbit and human colonic epithelial cells (Raqib et al., 2012). These antimicrobial peptides act as reservoirs for antimicrobial agents against a wide range of pathogens at the host-microbial interface (Zasloff, 2002; Heinemann et al., 2014). Notably, the administration of sodium butyrate to PE rats also improved the abundance of GM, particularly Firmicutes and Bacteroides, and significantly increased the levels of butyrate and valeric acid (Yong et al., 2022). This provides further evidence that SCFAs can improve inflammatory function and may help alleviate PE in pregnant rats.

### 5.2 Regulation of blood pressure by SCFAs

In a rat model utilizing spontaneously hypertensive rats (SHR), the GM is less diverse and richer, with a higher ratio of Firmicutes in contrast with Bacteroidetes (Ley et al., 2006; Gomez-Arango et al., 2016). Supplementation with acetate and propionate was observed to dramatically reduce blood pressure levels (Pluznick et al., 2013; Marques et al., 2017; Kim et al., 2018). In hypertensive patients, increased levels of LPS, intestinal fatty acid binding protein (I-FABP), and Th17 cells were observed, indicating heightened intestinal inflammation and permeability (Qi et al., 2017; Kim et al., 2018). Robles et al. found that, in SHR rats, alterations in gut microbiota contribute to elevated blood pressure by activating and accumulating T cells (Itani et al., 2016; Toral et al., 2019b). Furthermore, propionate has been found to significantly mitigate cardiac hypertrophy, fibrosis, and vascular dysfunction in hypertensive mice. It accomplishes this by reducing systemic inflammation, decreasing the frequency of splenic T cells, and suppressing Th17 cell activity (Bartolomaeus et al., 2019).

In SHR rats, compared to normal Wistar Kyoto rats (WKY rats), a decrease of approximately 30% in the T cell ratio was observed in the spleen, while an increase in the number of Treg cells in the intestinal lymph nodes and spleen was observed after treatment with probiotics or oral butyrate, which increased fecal butyrate content. These treatments also reduced Th17 cells to levels similar to those in the secondary lymphoid organs of WKY rats. These findings suggest that T cells play a crucial role in the hypertensive response triggered by fecal microbiota transplantation in SHR rats, and that SCFAs can reverse this effect (Robles-Vera et al., 2020).

In addition, bacteria that produce SCFAs may influence host blood pressure, either *via* direct vasodilatory effects or *via* plasminogen activator inhibitor-1 (PAI-1) (Pluznick, 2014; Li et al., 2022). Activation of GPR41 has been associated with the regulation of blood pressure. Butyric acid, an SCFA, can directly activate colonic vagal signal transduction through GPR41/43 receptors, leading to vasodilation and ultimately lowering blood pressure. GPR41 is expressed in various tissues, including the colon and blood vessels, and its activation by butyric acid promotes vasodilation and relaxation of smooth muscle cells, contributing to the reduction of systemic blood pressure (Onyszkiewicz et al., 2019). In addition, n-butyric acid increases the mRNA expression of PAI-1 in liver cells, and the enema of SCFAs stimulates increased rectal microcirculation (Gomez-Arango et al., 2016). In a meta-analysis, PAI-1 gene diversity was linked to an enhanced risk of PE (Morgan et al., 2013); the analysis found that pregnant women with hypertension, such as PE, showed increased placental PAI-1 expression and elevated levels of PAI-1 in their circulating blood. This is because hypertension can lead to vascular endothelial damage, triggering an inflammatory response, which in turn increases C-reactive protein and thus induces increased PAI-1 expression. Therefore, the inhibition of PAI-1 expression may inhibit the progression of hypertension. PAI-1 inhibition may also attenuate nitric oxide synthase-induced hypertension (Gomez-Arango et al., 2016). Therefore, we hypothesize that by manipulating the GM metabolites, SCFAs could reduce PAI-1 levels and thus alleviate hypertension in PE (**Figure 4**).

### 5.3 SCFAs may improve obesity and PE comorbidities such as diabetes

PE, as a metabolic disease, is influenced by factors such as obesity, diabetes, and other metabolic disorders, which are known to increase its prevalence (Pinheiro et al., 2019; Poon et al., 2019). SCFAs have been implicated in glucose and lipid metabolism as well as obesity (He et al., 2020; Litwin and Kułaga, 2021; Wu et al., 2021). They serve as an energy source and can activate intestinal gluconeogenesis, which is crucial for maintaining normal blood glucose levels and metabolic homeostasis (De Vadder et al., 2014; Machate et al., 2020). SCFAs also impact intestinal transit, function, and food intake by stimulating the release of peptide YY (PYY), an enteroendocrine hormone that reduces intestinal motility (Forbes et al., 2015), enhances glucose absorption in muscle and adipose tissue, and decreases food intake (Batterham et al., 2002; Van Den Hoek et al., 2004). Moreover, butyrate and propionate have been shown to inhibit the secretion of pro-inflammatory cytokines and chemokines in the placenta and visceral adipose tissue, improve insulin sensitivity, and regulate glucose uptake (Heimann et al., 2015; Roy et al., 2020). SCFAs also influence leptin secretion from adipocytes, thereby modulating food intake, body weight, and energy metabolism through the central nervous system (Sakakibara et al., 2006; Fujikawa et al., 2010; Wang et al., 2020; Alsharairi, 2021). Studies using non-pregnant animal models of obesity and diabetes have demonstrated that the inclusion of butyrate and propionate in the diet leads to reduced body weight, obesity, fasting glucose and insulin levels, improved insulin tolerance, and amelioration of diabetes symptoms (Gao et al., 2009; Henagan et al., 2015) (**Figure 4**).

In previous studies, GDM and PE were both common complications of pregnancy and had similar pathological changes, such as the elevated levels of pro-inflammatory cytokine TNF-αand IL-6, endothelial dysfunction, etc (Mcelwain et al., 2020; Phoswa and Khaliq, 2021), therefore there may be a correlation (Yang and Wu, 2022). For example, in a large international prospective Cohort study, it was found that after adjusting for age, BMI, urinary tract infection, OGTT, smoking and drinking status, family history and other factors, the occurrence of PE was still positively correlated with blood glucose level (Metzger et al., 2008). In term infants born to GDM women, GM, such as Bifidobacterium, is related to the decrease of SCFAs content in feces (Soderborg et al., 2020). Bifidobacterium can utilize human milk oligosaccharides (HMOs) through related degradation enzymes, thereby increasing the production of butyrate, propionate and other SCFAs (Gopalsamy et al., 2019; Alsharairi, 2023). The results of a control trial showed that butyrate could reduce intestinal inflammation in mice, and the expression of M2 macrophage related protein Arg1 was significantly increased. Then the treatment of M2 macrophage with butyrate increased the expression of H3K9/STAT6 signaling pathway (Hunter et al., 2010; Ji et al., 2016), which was related to reducing intestinal mucosal damage and the level of proinflammatory cytokines (Mosser and Edwards, 2008). In addition, the classic pathway of IL-4 inducing polarization of M2 Macrophage polarization involves STAT6 signaling pathway, which leads to the expression of Fizz1, Ym-1, Arg1 and other cytokines in macrophages (Mikita et al., 1996; Dong et al., 2011). SCFAs can also regulate the function of the Treg cells pool in the colon and prevent intestinal inflammation in mice in a GPR43 dependent manner (Furusawa et al., 2013; Smith et al., 2013). These results provide a molecular mechanism for SCFAs mediated protection against PE and comorbidities such as GDM, possibly by reducing intestinal inflammatory response.

## 6 Conclusions and recommendations

SCFAs, such as acetic acid, propionic acid, and butyric acid, are SCFAs, such as acetic acid, propionic acid, and butyric acid, are important metabolites of gut microbiota that play a significant role in immune homeostasis, particularly in the gut (Mell et al., 2015). They have potent anti-inflammatory actions and can enhance the function of Treg cells in the colon (Robles-Vera et al., 2020). Abnormal gut microbiota metabolism of SCFAs is associated with the pathogenesis of PE. Treating PE rats with sodium butyrate improved their gut microbiota composition, increased levels of butyric and valeric acid, and alleviated PE symptoms (Yong et al., 2022). Probiotic supplementation in the third trimester of pregnancy may reduce the risk of PE. Microecological regulators, including probiotics, prebiotics, and synbiotics, have shown promise in improving gut health and reducing PE incidence (Boe et al., 2013; Gomez-Arango et al., 2016). Prebiotics stimulate beneficial bacteria, such as Bifidobacterium and Colonococcus, which can help reduce blood pressure and glucose levels (Zhou and Xiao, 2018; Wu et al., 2021). Future, research should investigate the therapeutic role of special diets with probiotic supplements in preventing PE development (Huang et al., 2021). The correlation between GM dysregulation and PE highlights the potential for targeted treatments. Early detection and diagnosis of PE remain challenging, and further exploration of SCFAs and GM markers is warranted for more effective diagnosis and treatment.

While numerous microbiota environments, such as oral, gut, placental, and vaginal, are thought to contribute to PE, the role of GM in PE remains poorly understood. Moreover, evidence of a specific mechanism of GM in the gut is lacking. In addition to GM, other factors, such as dietary factors and daily sodium intake, can also influence the progression of PE. Therefore, a larger sample size and more detailed experiments should be conducted to eliminate other confounding factors and investigate the possible correlation between GM and PE progression.

A complete fecal sample includes bacteria, fungi, viruses, bacteriophages, and other metabolites from hosts and bacteria. To understand the role of different components in PE, it is necessary to employ multi-omics technologies, analyze deactivated fecal samples, and investigate specific bacterial metabolites. This approach can help identify critical components involved in PE development. Additionally, screening potential positive indicators and conducting pathway enrichment analysis can shed light on the regulatory metabolites in PE. Further research is needed to obtain detailed data on the microbiota composition of pregnant women following probiotic interventions. Determining the optimal dose and timing of probiotic interventions, such as before pregnancy, in the second trimester, or in the third trimester, requires more investigation. Addressing these questions will contribute to a better understanding of the effects of probiotics on GM and PE.

Further research is needed to identify the specific mechanisms by which SCFAs derived from GM act on different inflammatory cells in the context of PE. Longitudinal, multi-regional cohort studies are warranted to gain a comprehensive understanding of the microbial ecosystem in the treatment of PE and investigate the metabolic pathways involved. Functional experiments involving SCFAs and various cellular and animal models are necessary to observe phenotypic changes and elucidate the diverse effects and mechanisms of SCFAs at different stages of PE development. This research will help identify the specific SCFAs, optimal dosages, and intervention strategies that can effectively improve PE symptoms and provide valuable insights for clinical treatment.

The investigation of GM alteration and the role of SCFAs in the pathogenesis of PE holds promise for the prevention and treatment of this condition. However, further research is necessary to fully understand these relationships and validate the potential of probiotics or SCFAs as diagnostic and therapeutic approaches for PE. Large-scale studies involving diverse patient cohorts are needed to confirm the efficacy and safety of these interventions. Such endeavors will contribute to the development of innovative strategies for the prevention and treatment of PE.

## Author contributions

All authors contributed to data analysis, drafting, or revising the article, have agreed on the journal to which the article will be submitted, gave final approval of the version to be published, and agree to be accountable for all aspects of the work.
